# Effect of Hydrogen Plasma Treatment on the Sensitivity of ZnO Based Electrochemical Non-Enzymatic Biosensor

**DOI:** 10.3390/bios13080793

**Published:** 2023-08-07

**Authors:** Diana B. Tolubayeva, Lesya V. Gritsenko, Yevgeniya Y. Kedruk, Madi B. Aitzhanov, Renata R. Nemkayeva, Khabibulla A. Abdullin

**Affiliations:** 1 Faculty of Metallurgy and Mechanical Engineering, Karaganda Industrial University, Republic Ave. 30, Temirtau 101400, Kazakhstan; dianajianna@gmail.com; 2 Institute of Energy and Mechanical Engineering, Satbayev University, Satpayev Str., 22, Almaty 050013, Kazakhstan; y.kedruk@satbayev.university; 3 National Nanotechnology Laboratory of Open Type (NNLOT), Al-Farabi Kazakh National University, Al-Farabi Ave., 71, Almaty 050040, Kazakhstan; madi.aitzhanov@mail.ru (M.B.A.); nemkayeva.renata@gmail.com (R.R.N.)

**Keywords:** zinc oxide nanowires, hydrothermal route, non-enzymatic biosensors, ascorbic acid, air treatment, hydrogen plasma treatment, electrochemical properties

## Abstract

Information on vitamin C—ascorbic acid (AA)—content is important as it facilitates the provision of dietary advice and strategies for the prevention and treatment of conditions associated with AA deficiency or excess. The methods of determining AA content include chromatographic techniques, spectrophotometry, and electrochemical methods of analysis. In the present work, an electrochemical enzyme-free ascorbic acid sensor for a neutral medium has been developed. The sensor is based on zinc oxide nanowire (ZnO NW) arrays synthesized via low-temperature chemical deposition (Chemical Bath Deposition) on the surface of an ITO substrate. The sensitivity of the electrochemical enzyme-free sensor was found to be dependent on the process treatments. The AA sensitivity values measured in a neutral PBS electrolyte were found to be 73, 44, and 92 µA mM^−1^ cm^−2^ for the ZnO NW-based sensors of the pristine, air-annealed (AT), and air-annealed followed by hydrogen plasma treatment (AT+PT), respectively. The simple H-plasma treatment of ZnO nanowire arrays synthesized via low-temperature chemical deposition has been shown to be an effective process step to produce an enzyme-free sensor for biological molecules in a neutral electrolyte for applications in health care and biomedical safety.

## 1. Introduction

Modern medicine has demonstrated the importance of a wide class of substances, such as vitamins, minerals, and antioxidants, in maintaining basic physiological processes in the body and in the prevention and treatment of diseases. The development of simple methods of monitoring vitamin and nutrient levels is an important issue with respect to improving diagnostic tools when determining the quality of products, and it contributes to improving quality of life.

In particular, detection methods for vitamin C, otherwise known as ascorbic acid (AA), have attracted considerable research attention. Ascorbic acid is a water-soluble vitamin found in most biological systems, vegetables, and fruits [1]. Ascorbic acid plays an important role in human metabolism, namely, the removal of free radicals. The addition of ascorbic acid to a basic diet as an antioxidant in high doses has been used as an adjuvant therapy in the treatment of serious diseases such as cancer and Parkinson’s disease [2,3,4]. Ascorbic acid deficiency causes an increased likelihood of scurvy [5]. It is thus very important for medical applications and the food industry to determine the ascorbic acid content in natural and prepared foods, vegetables, fruit juices, medicines, and bodily fluids.

Various analytical methods, such as chemiluminescent, spectrometric, and luminescent methods, are being developed to determine ascorbic acid levels [6,7,8,9,10]. Most of these methods are based on the reducing properties of ascorbic acid, but the selectivity of these methods suffers due to the influence of other reducing agents in a sample [11]. Electrochemical methods are often used to detect ascorbic acid, as AA is an electroactive compound. The electrochemical method is characterized by high sensitivity, easy operation, and low cost [12,13].

The development of electrochemical biosensors based on thin films and zinc oxide ZnO nanostructures has been the subject of many studies [11,14,15,16,17,18,19]. The use of nanomaterials has modernized the signal transmission approach in biosensors, which has resulted in the improved sensitivity and performance of biosensors [20,21,22]. The effective use of zinc oxide in biosensors is possible due to high electrical conductivity, high exciton binding energy of 60 meV, and wide band gap of 3.37 eV [23]. Th wide band gap is able to support large electric fields, which provides high breakdown voltage and semiconductor stability [24].

Zinc-oxide-based biosensors have been recognized as promising due to their cost-effectiveness and non-toxicity, availability with respect to precursors, and high isoelectric point [25,26]. This high isoelectric point contributes to a high uptake of proteins, enzymes, and DNA due to electrostatic interactions [27]. These types of biosensors are often used to detect analyte types such as cancer cells, uric acid, cholesterol, ascorbic acid, and glucose [19,25,28,29,30]. ZnO is an important multifunctional nanomaterial and is used in optical and electrochemical biosensors [15,28].

Electrochemical biosensors are attractive devices with respect to analyzing the content of a biological sample due to their ability to directly convert a biological event into an electrical signal. The advantages of electrochemical biosensors include their miniaturisation, wide detection limits, simplicity, and reliability [31,32].

Two types of electrochemical sensors are used for the detection of ascorbic acid: enzymatic and non-enzymatic. The advantage of an electrochemical non-enzymatic sensor is that it has a high occurrence to the denaturation, retention, and extraction of enzyme-like catalysis at a negligible pH and temperature. Enzyme-free sensors used for the determination of ascorbic acid levels, which are of practical importance in both food processing and medical diagnostics [33,34,35,36,37], have attracted much research attention due to their high reproducibility and stability compared to enzyme-based sensors. However, since most enzyme-free sensors used for ascorbic acid rely on the chemical activity of the transition metal center, which can be easily altered, the fabrication of enzyme-free sensor elements for ascorbic acid detection with high performance remains a challenge [38]. The efficiency of sensors is evaluated using many parameters, such as speed, high sensitivity, stability, and operating voltage. Thus, a high-quality enzyme-free sensor for ascorbic acid determination must meet these conditions and must be related not only to the composition and surface properties of the sensor materials but also to their microstructures [39].

To achieve high-sensitivity ZnO-based electrochemical sensors, the presence of -OH groups in the electrolyte is necessary; therefore, ZnO electrochemical sensors most often use alkaline electrolytes. However, alkaline electrolytes lead to the dissolution of ZnO and the gradual degradation of the sensor. In addition, an alkaline environment is not the same as most natural environments. In neutral electrolytes at pH ~ 7, the sensitivity of ZnO electrochemical sensors inevitably decreases many times [25]. Therefore, it is necessary to develop methods for increasing the sensitivity of ZnO electrochemical sensors in such media.

The thermal treatment and hydrogen plasma treatment of zinc oxide semiconductor materials are common methods for changing their physicochemical properties [40,41,42]. Negatively charged oxygen forms adsorbed on the ZnO grain boundaries play the role of electron capture centers and form potential energy barriers, thereby reducing the Hall mobility and concentration of free carriers. During heat and hydrogen plasma treatments, these negatively charged forms of oxygen are desorbed, thus improving their electrical characteristics [23,43,44].

In the present study, we report a cost-effective, accurate, and highly sensitive non-enzymatic electrochemical biosensor of ascorbic acid based on ZnO NW using a neutral electrolyte based on PBS. To increase the sensitivity of the electrochemical biosensor based on zinc oxide nanowire layers, a method consisting of treating ZnO NW in hydrogen plasma was developed. The results revealed that thermal annealing in air followed by short-term treatment in hydrogen plasma significantly increases the sensitivity of the electrochemical ZnO NW biosensor.

## 2. Materials and Methods

### 2.1. Materials

Zinc nitrate hexahydrate Zn(NO_3_)_2_ × 6H_2_O (purity 98%), zinc acetate dihydrate (ZnAc_2_, (CH_3_COO)_2_Zn × 2H_2_O (purity 99.9%), hexamethylenetetramine (CH_2_)_6_N_4_ (HMTA, purity 99.9%), disodium hydrogen phosphate, Na_2_HPO_4_ × 12H_2_O (purity 98%), sodium dihydrogen phosphate NaH_2_PO_4_ × 2H_2_O (purity 98%), and L-ascorbic acid (purity 99%) were purchased from Sigma-Aldrich and used as received. All other used chemicals, such as ammonium (NH_4_OH) (28%), hydrogen peroxide (H_2_O_2_) (33%), and ethanol (C_2_H_5_OH), were of analytical grade. 

### 2.2. Synthesis of ZnO Nanowires

Samples with zinc oxide nanowires serving as an electrode for ascorbic acid detection were synthesized via low-temperature chemical deposition (Chemical Bath Deposition) [45] on glass ITO substrates (Sigma-Aldrich, St. Louis, MO, USA, resistance 8–12 Ohm/cm^2^) with an area of 1 cm^2^; the ITO layer thickness was 120–160 nm. Before synthesis, the substrates were thoroughly cleaned using piranha solution containing ammonia solution, hydrogen peroxide, and distilled water in a 1:1:4 ratio. Boiling the ITO–glass plates for 10 min in the piranha solution created a clean hydrophilic surface.

ITO-coated glass has good conductivity and chemical resistance and is less expensive than gold or glass carbon electrodes. In the first step, a ZnO seed layer was applied to the prepared substrates using the sol–gel method [23]. The sol solution was prepared by dissolving 0.4 g of zinc acetate in 10 mL of ethanol. This method is simple, cost-effective, efficient, and designed to create a high-quality ZnO seed layer for the subsequent growth of ordered nanostructures. Uniform distribution of the sol was achieved by spreading a few drops of the solution on the surface of a prepared substrate, which was fixed on a horizontal table, and then rotating it at a speed of ~2000 rpm for 5 min. The substrates were then dried in a desiccator at 120 °C for 20 min. Subsequent annealing at 450 °C for 60 min in a muffle furnace resulted in the formation of a uniform seed layer on the surface of the substrates.

The second stage involved the oriented growth of ZnO nanowires via low-temperature chemical deposition (Chemical Bath Deposition). The growth solution contained mixtures of aqueous solutions of 75 mM zinc nitrate and 75 mM hexamethylentetramine HMTA. The synthesis was carried out in a glass beaker for two hours at 90 °C in a water bath with a magnetic stirrer. The substrates with the seed layer were attached at a slight angle to the walls of the glass, with the back side facing the center of the glass. The beaker containing the working solution and the samples was sealed. At the end of the synthesis, the samples were extracted, washed with distilled water, and dried in a desiccator for 30 min at 115 °C. During this synthesis, the following chemical transformations occurred between zinc nitrate Zn(NO_3_)_2_ and HMTA [46]:

Zn(NO_3_)_2_ × 6H_2_O → Zn^2+^ + 2(NO_3_)^−^ + 6H_2_O,

C_6_H_12_N_4_ + 6H_2_O ↔ 6CHCHO + 4NH_3_,

NH_3_ + H_2_O ↔ NH_4_^+^ + OH^−^,

Zn^2+^ + 4(OH^−^) → Zn(OH)_4_^2−^ → ZnO(s) + H_2_O + 2(OH^−^),

Zn^2+^ + 2(OH^−^) → Zn(OH)_2_ → ZnO(s) + H_2_O.

In this synthesis, hydroxide ions are formed through the decomposition of HMTA and then react with Zn^2+^, thereby forming ZnO nanowires on the substrate seed layer.

Three types of samples were considered: initial (ZnO NW pristine), heat-annealed (ZnO NW AT), and heat-treated followed by hydrogen plasma treatment (ZnO NW AT+PT). During the heat treatment, the samples were placed in a muffle furnace at 450 °C for one hour. Treatment with hydrogen plasma (H-treatment) was carried out at room temperature in a quartz tube reactor with a diameter of 30 mm. The plasma was created using capacitive excitation with a frequency of 27.12 MHz, a power of about 15 W, and an in-reactor hydrogen pressure of ~50 Pa. The duration of the H-treatment was 3 min.

### 2.3. Characterization

The morphology of the synthesized ZnO NW samples was investigated using an electronic scanning microscope (SEM) with a direct-filament tungsten cathode (FEI, Hillsboro, OR, USA) Quanta 200i 3D. XRD measurements were carried out using an X-ray diffractometer X’pert MPD PRO (PANalitical, Almelo, The Netherlands). Raman measurements were taken using a Solver Spectrum spectrometer with a 473 nm solid-state exciting laser (NT-MDT, Zelenograd, Russia). The absorption and transmittance spectra were measured with a Lambda 35 UV-vis spectrophotometer (PerkinElmer, Waltham, MA, USA). The PL spectra were recorded using a Cary Eclipse spectrofluorimeter (Agilent, Santa Clara, CA, USA) under 300 nm excitation at room temperature. XPS spectra were analyzed using a NEXSA X-ray Photoelectron Spectrometer (Thermo Scientific, Waltham, MA, USA). The electrochemical characteristics were determined via cyclic voltammetry (CV). The measurements were performed in 0.1 M of phosphate-buffered saline (PBS) (pH = 7) at room temperature using a Corrtest CS310 single-channel potentiostat/galvanostat and a three-electrode electrochemical cell containing an auxiliary platinum electrode and an Ag/AgCl chlorosilver reference electrode along with the working ZnO electrode. The concentration of AA in the phosphate-buffered solution varied from 0.3 mM to 3 mM.

## 3. Results

### 3.1. Morphology

**Figure 1:**
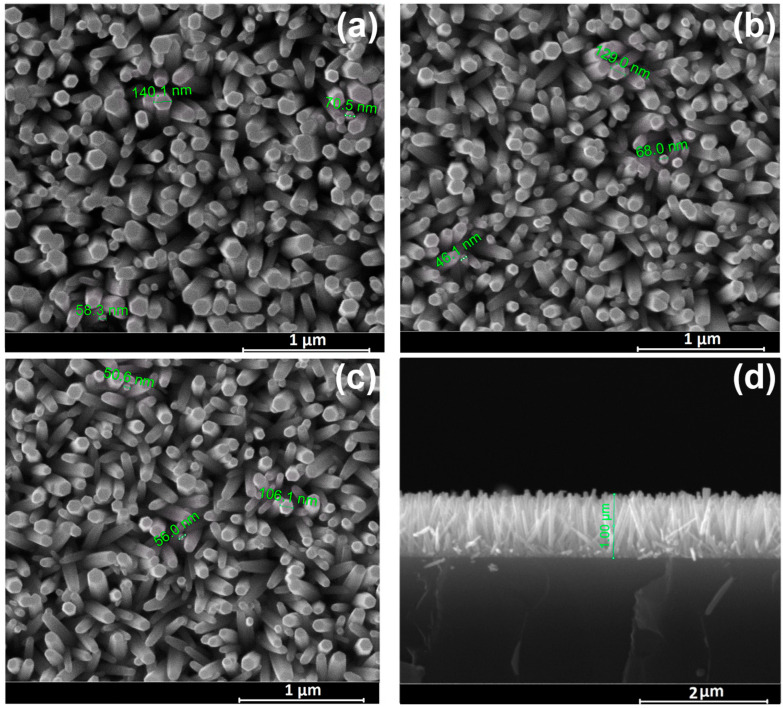
SEM images of ZnO nanowire arrays samples: (**a**) Pristine ZnO (top view); (**b**) ZnO subjected to thermal treatment (AT) (top view); (**c**) ZnO subjected to thermal treatment followed by hydrogen plasma treatment (AT+PT) (top view); (**d**) Pristine ZnO (cross section).

The uniform application of a ZnO seed layer to the ITO substrate ensured the high nanowire density and homogeneity of the growing layer. Figure 1 shows SEM images of all the considered samples, as pristine (Figure 1a), heat treated (Figure 1b) as well as subjected to thermal treatment followed by hydrogen plasma treatment (Figure 1c). Zinc oxide has a hexagonal wurtzite structure consisting of a non-polar hexagonal plane surface and a positively charged polar surface (0001). During the synthesis process, the negatively charged Zn(OH)_4_^2−^ ions are first attracted to the positively charged surface (0001), and this is followed by crystal growth along the c-axis. The chelating agent, hexamethylenetetramine, adsorbs to the non-polar surface, thereby promoting the growth of nanowires along the c-axis.

The SEM images show that the freshly prepared and processed samples consist of ZnO nanowires with a hexagonal cross-section, presenting a flat end and a diameter of 50–100 nm, oriented perpendicular to the substrate’s surface (Figure 1d). The layer thickness for all samples was about 1 µm. It can be seen from the figures that the morphology of the ZnO nanowires did not change after heat treatment (Figure 1b) subsequent H-plasma treatment (Figure 1c).

### 3.2. Structural Properties

**Figure 2:**
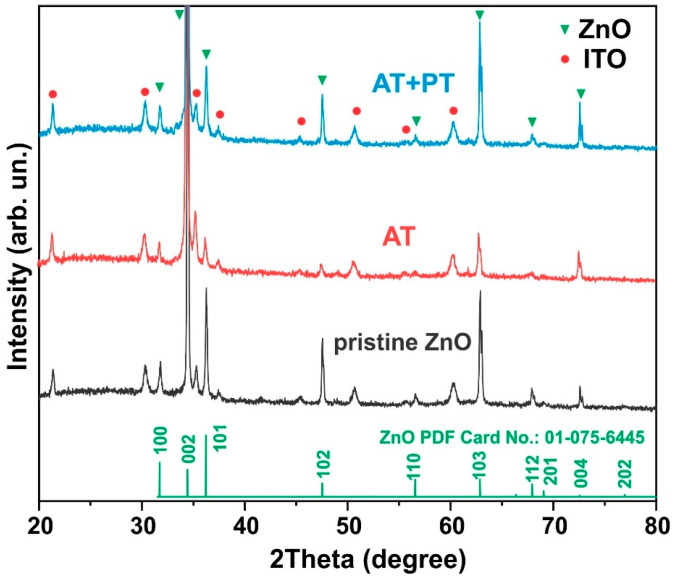
XRD patterns of ZnO nanowire arrays (pristine ZnO, AT, and AT+PT samples) on ITO substrates.

X-ray diffraction analysis of the examined ZnO samples (Figure 2) showed that the diffractograms of both the pristine sample and the ZnO NW AT and ZnO NW AT+PT samples reveal Bragg reflexes of hexagonal ZnO (JCPDS 01-075-6445) as well as ITO layer reflexes. All the samples exhibited sharp and strong diffraction at 2θ = 34.44°, indicating that the arrays of the ZnO nanowires were predominantly perpendicular to the substrate’s surface, with the nanowire axis oriented along the (002) direction, which is consistent with the SEM results (Figure 1). Estimates of the transverse dimensions of the ZnO crystallites along the (002) plane were made using the Scherrer formula L = 0.9 λ/β cos θ, where θ is the diffraction angle, λ is the X-ray wavelength, and β is the reflection width at half-height. The typical half-width of the corresponding reflex was ~0.14 (in units of 2θ), which corresponds to transverse dimensions of nanowires with a size of ~50–100 nm and is consistent with the results obtained using scanning electron microscopy (Figure 1).

Notably, no significant changes in the positions and sizes of the peaks in the diffractograms after the thermal and H-plasma treatments were observed, indicating that the basic elemental atomic structure of the material was maintained during the treatments used and that the samples were highly crystalline.

### 3.3. Raman Spectra

**Figure 3:**
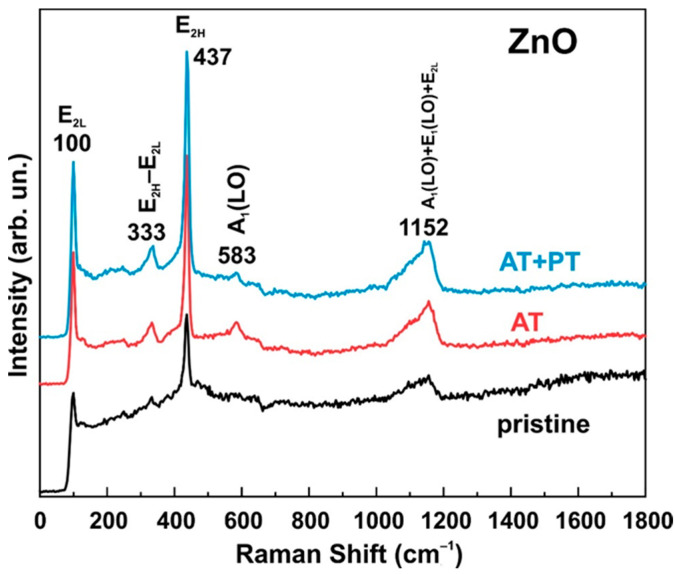
Raman spectra of ZnO nanowire arrays (pristine ZnO, AT, and AT+PT samples).

Raman spectra were measured for all three sample types (pristine ZnO, AT and, AT+PT samples) (Figure 3). As shown in the presented spectra, the E_2L_ mode was observed at 100 cm^−1^. This mode is characteristic of the Raman spectra of ZnO and is associated with the motion of the oxygen and zinc sublattice in the wurtzite structure of the oxide. The E_2H_ mode observed at 437 cm^−1^ has a high intensity in the Raman spectrum, which confirms the high crystallinity of the samples. In addition, we can also observe another optical phonon mode near symmetry A1 located at 333 cm^−1^, which corresponds to a vibrational mode (E_2H_–E_2L_) [47]. The A_1_(LO) mode was observed at 583 cm^−1^. The broad peak at 1152 cm^−1^ can be ascribed to the occurrence of a multiphonon process (A_1_(LO) + E_1_(LO) + E_2L_) [48]. 

It should be noted that the main difference between the Raman spectra of the considered series of samples lies in the fact that the thermal treatment in an atmosphere at 450 °C and subsequent treatment in hydrogen plasma led to an increase in the intensity of the vibrational modes whose peaks fall at 100 cm^−1^, 333 cm^−1^, 437 cm^−1^, and 1152 cm^−1^. The increase in the intensities of these modes and the absence of new peaks after the thermal and H-treatments indicate an increase in the crystallinity degree of the considered ZnO-oxide samples, thereby confirming the results presented in the XRD analysis data.

In addition, the position of the E_2H_ mode is sensitive to the stress along the oxide structure. A shift in the wave number towards higher values indicates the presence of compressive stresses along the structure, while a shift in the wave number towards lower values reflects tensile stress. The unchanged position of the 437 cm^−1^ peak following the treatments used excludes the presence of these effects along the zinc oxide structure. The A_1_(LO) mode, which is often associated with defects in the ZnO structure [49], becomes less pronounced in the samples annealed in an atmosphere at 450 °C for one hour followed by treatment in hydrogen plasma, which also indicates the high crystalline quality of the ZnO NW samples after the treatments had been performed.

### 3.4. Optical Properties

Figure S1 (Supplementary Materials) shows the optical density spectra of the considered ZnO nanowire samples measured at room temperature. It can be seen that all the samples absorb in the UV range of 300–380 nm and have weak absorption in the visible region. Based on the optical density spectra, it is possible to estimate the optical band gap width from the Tauc relation for the absorption edge [26]. The plot of the dependence (αhν)^2^ on hν is given in Figure S1, where the extrapolation of the linear parts of the curves to the energy axis allows for the estimation of the forbidden zone energy of the samples, which, for all samples, is ~3.31 eV, which is close to the table value. The error in the estimation of the optical Eg was ~3.5%. The transmission spectra presented in Figure S1 show that all the samples are transparent in the visible range. The ZnO NW AT+PT sample has higher transmittance, which can be attributed to both the passivation of defects in these samples and plasma cleaning during treatment.

### 3.5. Photoluminescence Spectra

**Figure 4:**
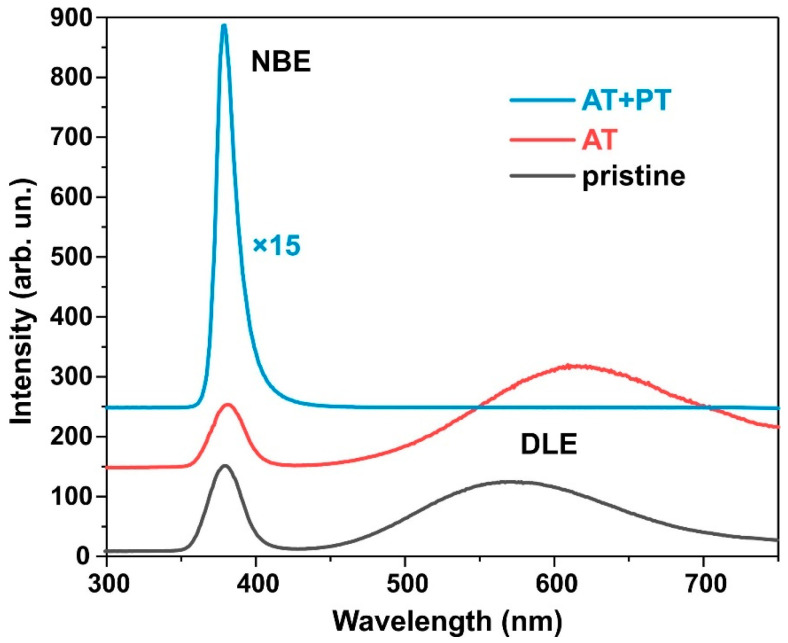
Photoluminescence spectra of ZnO nanowires.

Figure 4 shows the photoluminescence (PL) spectra measured at room temperature of the pristine synthesized ZnO NW samples as well as those annealed at 450 °C for one hour and subjected to hydrogen plasma treatment after thermal annealing. In all the samples, a narrow band of UV radiation at 380 nm (3.27 eV) is present, which was a result of the PL intensity near the band edge (the near band edge emission, NBE) due to the recombination of free excitons during exciton–exciton collisions. In addition, the pristine sample and the heat-treated sample have a broad band in the spectral range of 450–700 nm, which, in the literature, is commonly associated with defects in ZnO such as oxygen vacancies VO or zinc vacancies VZn (deep-level emission DLE) [50,51].

In the pristine samples, the DLE peak corresponded to the 570 nm (~2.2 eV) yellow and green region. Thermal annealing led to a decrease in intrinsic PL intensity near NBE. At the same time, after the thermal treatment, the PL intensity at transitions through deep DLE levels increased, and the peak shifted to the orange–red region at 620 nm (~2.0 eV). Orange emission is usually observed in oxygen-rich systems. It is attributed to either interstitial oxygen atoms (Oi) due to the excess of oxygen on the ZnO surface or to hydroxyl groups (OH) [52]. The DLE FL peak at 620 nm indicates that the oxygen inter-nodes in these samples were the dominant defects. The red and yellow band of PL is attributed to transitions of electrons from the conduction band to oxygen inter-nodes located 1.95–2.14 eV below the conduction band [53]. 

Plasma treatment in a hydrogen atmosphere significantly increases the NBE intensity and completely passivates the DLE band. As shown in [23], the NBE intensity after plasma treatment in hydrogen increases with the amount of adsorbed oxygen on the ZnO’s surface during preliminary annealing. Air annealing at 450 °C followed by plasma treatment increases the NBE intensity of PL by a factor of 72 compared to the NBE intensity of the pristine samples. Previous studies [23,44] noted that H-treatment passivates the surface states created by oxygen adsorbed at the grain interfaces during air pre-annealing, which are responsible for the increase in NBE PL. Thus, it can be seen from the PL spectra that the annealing of ZnO nanowires followed by plasma treatment not only purifies the material produced by moisture and OH ions but also affects various optical recombination channels. The testing of the electrical resistivity of the ZnO layers using a two-probe method also showed that the layer resistivity drops from hundreds of kiloohms in pristine ZnO to less than 100 ohms in the ZnO AT+PT samples.

### 3.6. XPS Spectra

**Figure 5:**
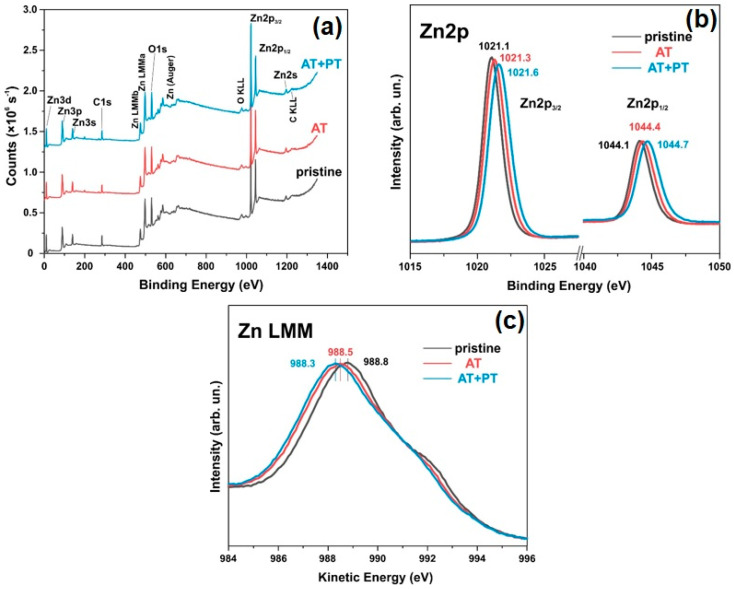
XPS spectra of pristine ZnO, AT, and AT+PT samples: (**a**) Overall XPS survey spectra; (**b**) Zn2p_1/2_ and 2p_3/2_ core-level scans; (**c**) Zn LMM Auger spectra.

**Figure 6:**
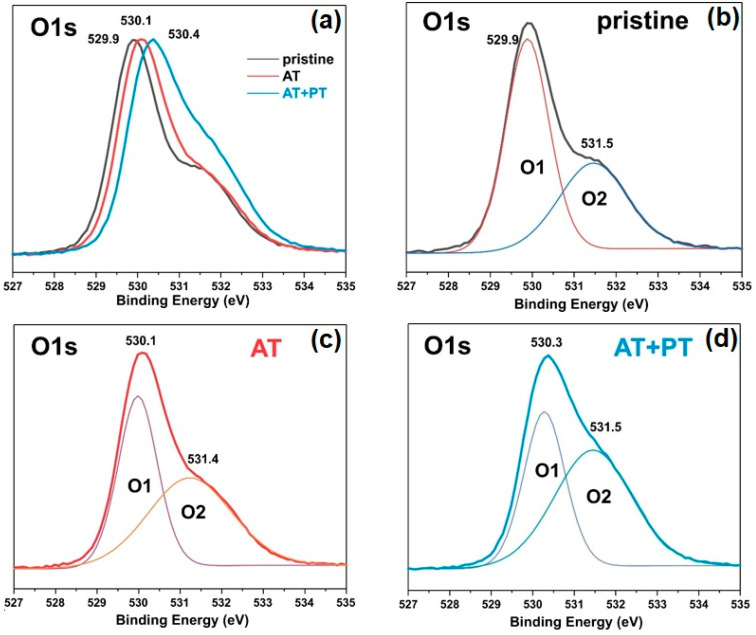
XPS spectra of pristine ZnO, AT, and AT+PT samples: (**a**) XPS core-level scan of O1s; (**b**–**d**) deconvolution of XPS core-level scan of O1s spectra of pristine ZnO NW, AT, and AT+PT samples, respectively. Gaussian’s lines Q1 and Q2 demonstrate the composition into individual components.

The elemental composition of the surfaces and the chemical states of the pristine ZnO sample and the AT and AT+PT samples were investigated using XPS spectroscopy (Figure 5 and Figure 6). An overview of the XPS spectra of the ZnO NW samples is shown in Figure 6a. These spectra confirm the presence of Zn and O in the samples. Figure 5b shows the high-resolution XPS spectra of Zn2p, with the doublet peaks Zn2p_3/2_ and Zn2p_1/2_ corresponding to the oxidation degree of Zn^2+^ (Zn-O). The distance between the peaks is ~23 eV, which corresponds to the spin-orbit splitting value of Zn2p ZnO [54]. The Zn2p peaks are often accompanied by an Auger peak at a kinetic energy value of ~990 eV.

The asymmetric XPS spectra of O1s allowed us to deconvolve the peaks of the spectra into two Gaussian peaks, namely, O1 and O2, at binding energies of 529.88 eV and 531.48 eV, for the pristine ZnO sample (Figure 6b). The peak at 529.9 eV characterizes the degree of oxidation of the O2 lattice of ZnO wurtzite (O1). The intensity of this peak indicates the number of oxygen atoms in the hexagonal structure of ZnO wurtzite. The O2 peak at 531.5 eV corresponds to oxygen vacancies or defects in the crystal lattice (OV) as well as the presence of OH bonds [55]. These XPS results are in agreement with the data from the literature regarding ZnO [55,56]. The O1 peak for the AT and AT+PT samples was shifted to higher energies (Figure 6c,d). The relative intensity of the O2 peak of the ZnO NW AT+PT sample increased, which might have been due to the formation of OH bonds as H-treatment causes reduction processes. An analysis of Figure 5 and Figure 6 reveals that the H-treatment brough about a change in the XPS spectra of the ZnO NW samples. The pristine ZnO sample synthesized via the low-temperature hydrothermal method has a high concentration of surface defects. The thermal treatment of the pristine samples reduced the concentration of defects and transformed them into more stable configurations. H-treatment with preliminary annealing in air reduced the concentration of passivated states, which was also confirmed by the significant differences in the photoluminescence spectra of the considered series of samples (Figure 4).

### 3.7. Biosensors’ Characterization

**Figure 7:**
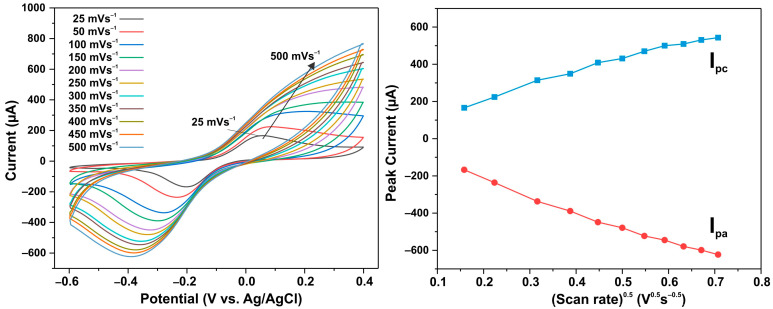
(**a**) Cyclic voltammograms of pristine ZnO NW electrode in PBS solution (0.1 M, pH = 7) at different scan rates within 25–500 mV s^−1^; (**b**) graph of redox peak current versus square root of scan rate.

An electrochemical non-enzymatic determination of ascorbic acid (AA) was carried out, for which ZnO NW arrays grown on a conductive ITO layer were used as a working electrode. In order to elucidate the mechanism of AA’s reaction with the electrode surface, the variation in the AA oxidation current peaks on the ITO/ZnO electrode was analyzed at different scanning speeds ranging from 25 to 500 mV/s. Figure 7a presents cyclic voltammetry diagrams showing the effect of different scanning rates of 25–500 mV/s on the electrochemical response of the electrode with the pristine ZnO NW sample in a 0.1 M buffer solution with 0.1 mM of AA. Obviously, the anodic and cathodic peak potentials shift to the positive and negative sides, respectively, when the scanning speed increases, which is a sign of AA’s diffusion mechanism acting on the ITO/ZnO electrode surface. At ~0.2 V, maximal oxidation currents are observed, while at ~−0.4 V, maximal reduction currents are observed. A linear dependence of the peaks of the oxidation currents I_pc_ and reduction currents I_pa_ on the square root of the scanning speed was observed, which also indicates a diffusion mechanism consisting of AA’s reaction with the electrode surface.

The electrochemical sensor ZnO functions on the basis of redox reactions occurring on its surface. Ascorbic acid exhibits reducing properties, so when AA comes into contact with the surface of the ZnO sensor, a redox reaction takes place. In enzyme-free electrooxidation, the AA molecule gives up electrons to the ZnO surface and is oxidized to dehydroascorbic acid and H_2_O_2_ in the presence of oxygen; this is followed by the release of electrons from H_2_O_2_ after its conversion to oxygen and protons [36]. This reaction caused by these released electrons leads to the generation of an electrical signal [57]. The intensity of the response depends primarily on the concentration of AA, the specific area of the sensor, and the redox reaction activity of the semiconductor sensor’s surface. Meanwhile, surface activity and sensitivity to ascorbic acid can be enhanced by modifying the surface with specific functional groups, heterogeneous nanoparticles, etc., to increase binding affinity to ascorbic acid molecules. 

**Figure 8:**
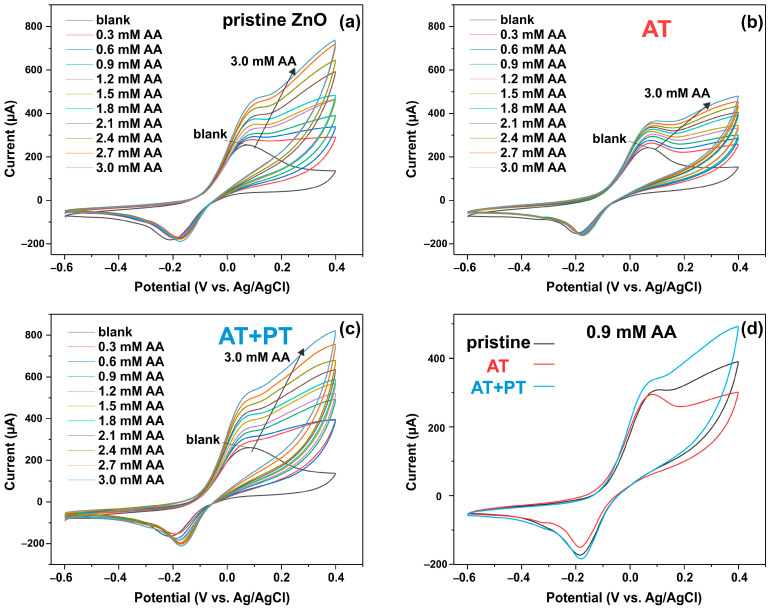
CV response of ZnO NW/ITO electrode vs. AA concentration: (**a**) pristine ZnO sample; (**b**) AT sample; (**c**) AT+PT sample; (**d**) CV curves of pristine ZnO, AT, and AT+PT samples at AA concentration of 0.9 mM.

**Figure 9:**
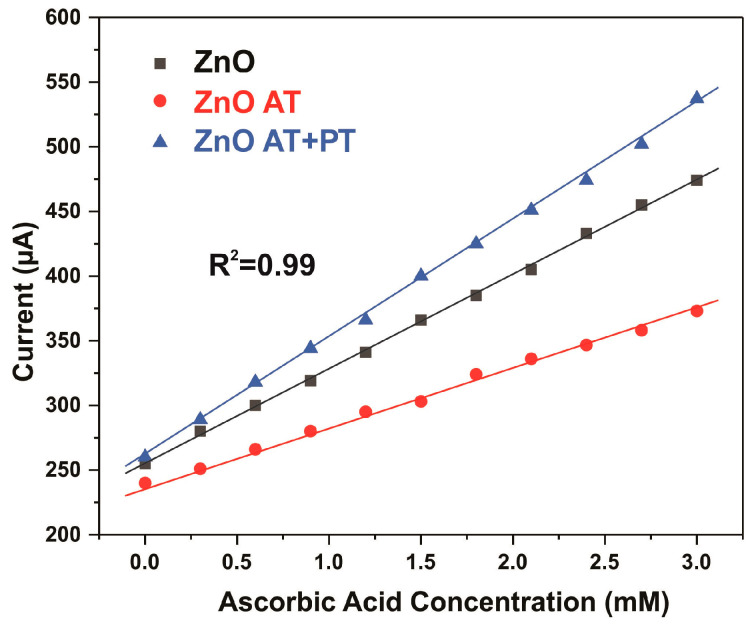
Calibration plot of CV peak current at various AA concentrations in 0.1 M of PBS and at a scan rate of 25 mV s^−1^.

Figure 8a–c show cyclic voltammetry diagrams for the three types of synthesized ZnO NW samples at different AA concentrations ((0.3 ÷ 3) mM) in PBS buffer solution at a scan rate of 25 mV/s. It can be seen that by adding AA, the current response of the ZnO NW/ITO electrode enhances the redox reaction of AA with the analyte. Figure 8d shows the CV curves of the pristine ZnO, AT, and AT+PT samples at an AA concentration of 0.9 mM to compare the sensitivity of the samples. The calibration plot for the ZnO NW/ITO samples is plotted for the oxidation current peaks as a function of AA concentration (Figure 9).

It was observed that there was a linear relationship between the peak oxidation current I_pc_ and the AA level in 0.1 M PBS with a correlation coefficient of R^2^ = 0.99 for all samples. The sensitivity of the prepared ZnO NW/ITO electrodes S was determined from the ratio of the slope of the calibration curves m to the active surface area of electrode A. The sensitivity values for AA calculated from the slope of the calibration curves measured in neutral PBS electrolyte were 73, 44, and 92 µA mM^−1^ cm^−2^ for the ZnO-NW-based sensors, AT, and AT+PT, respectively. The ZnO NW samples subjected to thermal annealing followed by treatment in hydrogen plasma had the highest sensitivity in the considered sample series.

To obtain complete information on the charge transfer efficiency and charge separation at the ZnO NW/ITO interface, electrochemical impedance spectroscopy (EIS electrochemical impedance) measurements were carried out in the range of 0.1÷10^5^ Hz at a bias voltage of +0.1 V. Figure S2a (Supplementary Materials) shows the Nyquist plot between the imaginary and actual impedance for ZnO NW, ZnO NW AT, and ZnO NW AT+PT in 0.1 M PBS. The arc radius characterizes the resistivity of the boundary layer on the electrode surface of ZnO NW/ITO [58]. The inset in Figure S2a shows an equivalent Randle circuit where R1 is the equivalent series resistance of the electrolyte solution, R2 is the charge transfer resistance, W_s_ is the Warburg impedance, and CPE (constant phase element) is the circuit element used to describe the capacitance exhibited in real electrochemical systems due to surface roughness or reaction rate distribution. The R2 values were 2382 kΩ (ZnO AT), 1610 kΩ (pristine ZnO), and 801 kΩ (ZnO AT+PT). It was observed that the R2 of pristine ZnO was less than the R2 of ZnO AT, and the R2 of ZnO AT+PT was less than the R2 of pristine ZnO. This finding indicates that H-treatment following thermal annealing facilitates electron transfer from the ZnO nanowires to the ITO electrode.

## 4. Discussion

In the study of the photoluminescence properties of the considered samples (Figure 4), it was noted that the treatment of the synthesized ZnO NW arrays in an atmosphere at 450 °C for an hour followed by short-term treatment in hydrogen plasma resulted in the passivation of surface states created by oxygen adsorbed at the grain boundaries during preliminary annealing in air. It was shown in [23,44,59] that this results in a decrease in the resistivity of the zinc oxide layers. The main reason for the resistivity decrease is the increase in the mobility of charge carriers as a result of scattering reduction at the grain boundaries, which, in turn, is a result of the hydrogen passivation of the acceptor traps on the ZnO polycrystals’ surfaces [44,58].

The analyses of the XPS and Auger spectra show that the thermal and plasma treatments resulted in a shift in the Auger peak to lower energies (Figure 5c), while, simultaneously, the Zn2p_3/2_ and Zn2p_1/2_ peaks shifted to higher energies (Figure 5b), indicating that in the ZnO NW AT+PT samples, the valence electron cloud density of the Zn and O surfaces decreases, and the binding energy of the valence electron and the electron residual level increases. The intensity of the peaks decreased after heat treatment. The decrease in the peak intensities might have been due to the surface potential [60], as indicated by the shift in the oxygen lines after the high resolution XPS treatments used for the O element (Figure 6a). The increase in the intensity of the oxygen O2 band, which corresponds to non-lattice O^2−^ ions or O^2−^ ions in oxygen vacancies, is consistent with an increase in the concentration of free carriers in the ZnO AT+PT samples. In other words, the concentration of recombination centers in the ZnO AT+PT samples decreased after H-treatment. This is also indicated by the optical transmission spectra, which showed that E_g_ is slightly increased by heat treatment and that plasma treatment leads to the recovery of E_g_. H-plasma treatment cleaned the material of moisture and OH^−^ ions, affected various optical recombination channels, and increased the concentration of passivated states. This led to surface activation and an increase in prevalence of the role of surface reactions with the analyte, i.e., increasing the sensitivity of the sensor.

In electrochemical measurements, redox reactions take place in a solution at the electrode–electrolyte interface, during which most of the chemical energy is converted into electrical energy. The main stage of the electrochemical process is the exchange of electrons, ions, and electron vacancies between the electrode and the solution. The decreased resistivity of the ZnO NW AT+PT samples increased the number of electrons involved in the oxidation process when interacting with AA molecules, thereby increasing the oxidation peaks and precipitating the higher sensitivity of this electrode compared to the pristine ZnO and ZnO AT samples (Figure 9).

A decrease in charge transfer efficiency leads to an increase in the arc radius on the Nyquist plot and an increase in the resistance of the ZnO layer of the working electrode. As can be seen from Figure S2a (Supplementary materials), the treatment of the synthesized ZnO NW layers in hydrogen plasma with preliminary annealing in an atmosphere decreased the surface layer resistance of the working electrode, indicating an increase in charge transfer efficiency and the interfacial separation of charge carriers on the ZnO surface. The analysis of the frequency dependences of the active and reactive impedance of the considered samples (Figure S2b,c) in the low-frequency region also revealed that after thermal treatment in air, the impedance of the synthesized samples increased, and the ZnO AT+PT samples presented the lowest value. These results prove that H-plasma treatment with a preliminary thermal treatment of samples in air contributes to an increase in the number of free electron carriers, which accelerates the charge transfer and reduces impedance, which, in turn, increases the sensitivity of the ZnO AT+PT samples, thus constituting findings consistent with the results presented above.

Table 1 shows the sensory characteristics of the different ascorbic acid detection electrodes measured using different techniques, namely, differential pulse voltammetry (DPV) and cyclic voltammetry (CV).

Compared to other studies, the ZnO NW/ITO electrode proposed in this work, which was based on ZnO nanowire arrays subjected to thermal treatment followed by H-plasma treatment, shows a high sensitivity of 92 µA mM^−1^ cm^−2^ and is thus a suitable material for the development of an analytical device for AA detection in the medical diagnostics and food industries.

**Figure 10:**
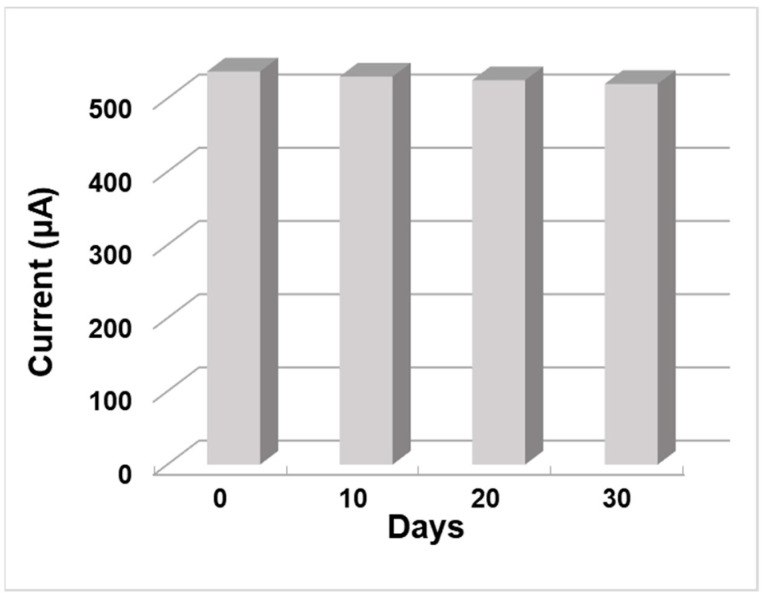
The stability of ZnO NW (AT+PT)/ITO electrode current response in 3 mM AA solution over 30 days.

The stability and lifetime of the ZnO NW/ITO electrode whose samples were subjected to thermal annealing followed by H-plasma treatment were evaluated using CV response measurements in 3 mM AA solution for 30 days. The synthesized ZnO NW AT+PT samples were stored in a dry state in air and at room temperature. As can be seen from Figure 9, the ZnO NW/ITO electrode retained 98.7% of its initial response after 10 days, 97.8% after 20 days, and 96.8% after 30 days, indicating the high stability of these ZnO nanowire layers. In [44], it was shown that the H-treatment of ZnO samples with pre-annealing in an atmosphere contributes to surface stabilization, resulting in ZnO NW AT+PT showing no appreciable ageing effect, as seen in Figure 10.

## 5. Conclusions

The present study shows that zinc oxide nanowire arrays grown using a low-temperature hydrothermal method are efficient, cost-effective, and reliable enzyme-free ascorbic acid biosensors with stable parameters. A simple method for increasing the sensitivity of ZnO sensors through thermal treatment followed by H-plasma treatment was developed, and stable and efficient ZnO NW/ITO electrodes presenting a high sensitivity of 92 µA mM^−1^ cm^−2^ were synthesized. The prepared electrodes represent promising candidates for use in non-enzymatic sensors for AA detection in the medical diagnostics and food industries.

## Figures and Tables

**Table 1:** Comparison of AA determinations in various samples achieved using electrochemical methods.

Type of Electrode	Technique	Sensitivity, µA mM^−1^ cm^−2^	Reference
Mn-doped ZnONRs/GO/GCE	DPV	0.169	[61]
ZnO nanorods	CV	32	[62]
ZnO-CuxO-PPy	CV	47.32	[63]
ANF-C700	DPV	1.06	[64]
APM/CNTPE	DPV	60.3	[65]
ZnO	CV	73	This article
ZnO AT	CV	44	This article
ZnO AT+PT	CV	92	This article

## Data Availability

Not applicable.

## Supplementary Materials

The following supporting information can be downloaded at: https://www.mdpi.com/article/10.3390/bios13080793/s1, Figure S1: UV–visible absorption (a) and transmittance (b) studies of ZnO nanowires.; Figure S2: (a) Nyquist semicircle graph for fabricated ZnO NW/ITO electrodes; (b) dependences of the active impedance components on frequency; (c) dependences of the reactive impedance components on frequency.
