# Supplementary material for: Effect of Hydrogen Plasma Treatment on the Sensitivity of ZnO Based Electrochemical Non-Enzymatic Biosensor
Source: Biosensors (Basel). 2023 Aug 7;13(8):793. doi: 10.3390/bios13080793 (PMC10452905; doi:10.3390/bios13080793)
Supplement: Supplementary file 1 [file biosensors-13-00793-s001.zip › biosensors-2532775-supplementary.pdf]

biosensors

Supplementary materials

Effect of Hydrogen Plasma Treatment on the Sensitivity of ZnO based Electrochemical Non-Enzymatic Biosensor

Diana B. Tolubayeva [1], Lesya V. Gritsenko [2,3,*], Yevgeniya Y. Kedruk [2,3], Madi B. Aitzhanov [3], Renata R. Nemkayeva [3], and Khabibulla A. Abdullin [3,*]

[1] Karaganda Industrial University, Republic Ave. 30, Temirtau 101400, Kazakhstan; dianajianna@gmail.com
[2] Satbayev University, Satpayev str., 22, Almaty 050013, Kazakhstan; y.kedruk@satbayev.university (Y.Y.K.)
[3] National Nanotechnology Laboratory of Open Type (NNLOT), al-Farabi Kazakh National University, al-Farabi ave., 71, Almaty 050040, Kazakhstan; madi.aitzhanov@mail.ru (M.B.A.); nemkayeva.renata@gmail.com (R.R.N.)
* Correspondence: l.gritsenko@satbayev.university (L.V.G.), kh.abdullin@physics.kz (K.A.A.)

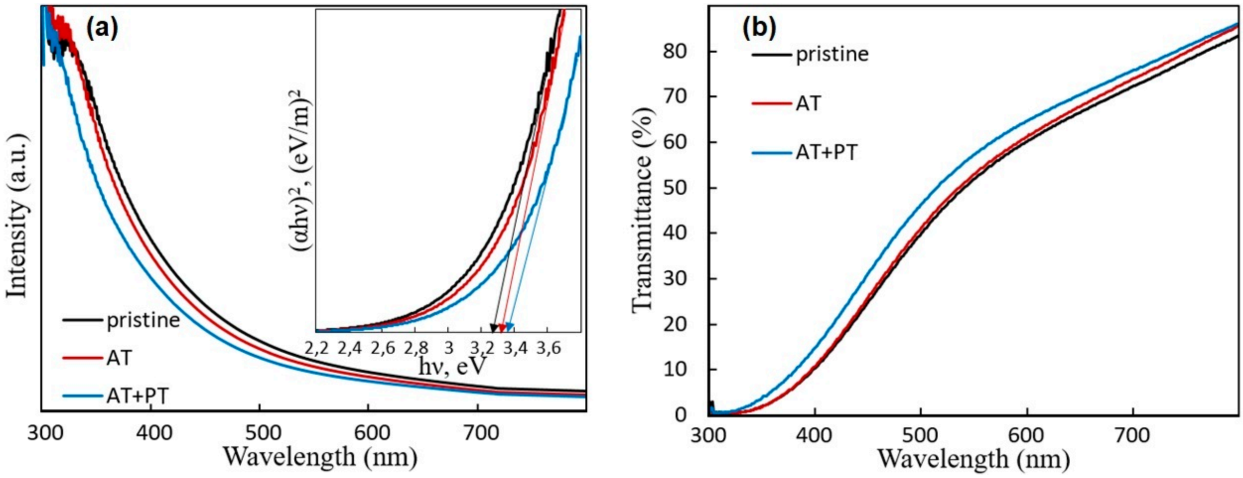

Figure S1. UV-Visible absorption (a) and transmittance (b) studies of ZnO nanowires.

Citation: To be added by editorial staff during production.

Academic Editor: Firstname Lastname

Received: date
Revised: date
Accepted: date
Published: date

Copyright: © 2023 by the authors. Submitted for possible open access publication under the terms and conditions of the Creative Commons Attribution (CC BY) license (https://creativecommons.org/licenses/by/4.0/).

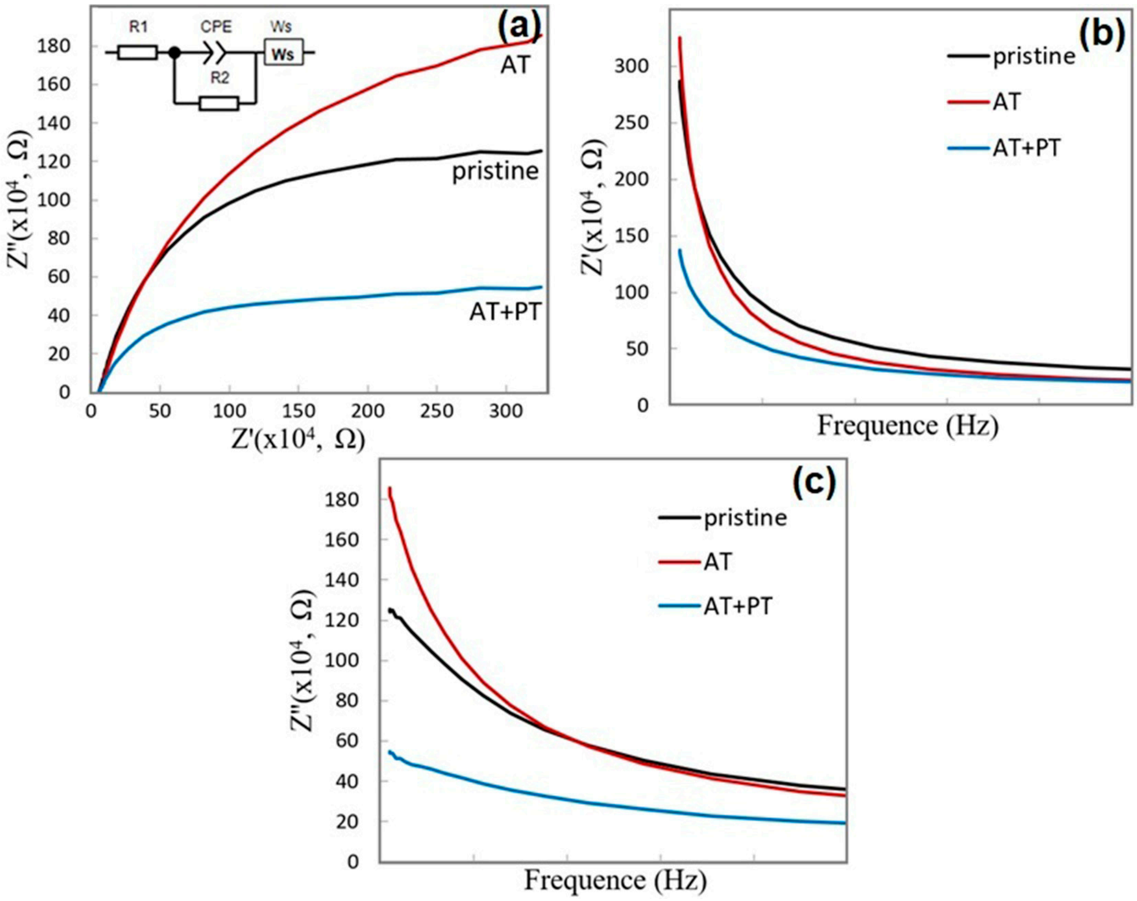

Figure S2. (a) Nyquist semicircle graph for fabricated ZnO NW/ITO electrodes; (b) dependences of the active impedance components on the frequency; (c) dependences of the reactive impedance components on the frequency.
